# Peripheral and central changes induced by neural mobilization in animal models of neuropathic pain: a systematic review

**DOI:** 10.3389/fneur.2023.1289361

**Published:** 2024-01-05

**Authors:** Federico Salniccia, Silvia de Vidania, Leticia Martinez-Caro

**Affiliations:** ^1^Faculty of Sport Sciences, Universidad Europea de Madrid, Villaviciosa de Odón, Spain; ^2^Facultad de Ciencias de la Salud, Universidad Internacional de La Rioja, Logroño, Spain; ^3^Facultad de Ciencias Sociales Aplicadas y de la Comunicación, UNIE Universidad y Empresa, Madrid, Spain

**Keywords:** physical therapy, animal models, neural mobilization, biomarkers, central nervous system, peripheral nervous system

## Abstract

**Introduction:**

Neural mobilization (NM) is a physiotherapy technique involving the passive mobilization of limb nerve structures with the aim to attempt to restore normal movement and structural properties. In recent years, human studies have shown pain relief in various neuropathic diseases and other pathologies as a result of this technique. Improvement in the range of motion (ROM), muscle strength and endurance, limb function, and postural control were considered beneficial effects of NM. To determine which systems generate these effects, it is necessary to conduct studies using animal models. The objective of this study was to gather information on the physiological effects of NM on the peripheral and central nervous systems (PNS and CNS) in animal models.

**Methods:**

The search was performed in Medline, Pubmed and Web of Science and included 8 studies according to the inclusion criteria.

**Results:**

The physiological effects found in the nervous system included the analgesic, particularly the endogenous opioid pathway, the inflammatory, by modulation of cytokines, and the immune system.

**Conclusion:**

On the basis of these results, we can conclude that NM physiologically modifies the peripheral and central nervous systems in animal models.

## 1 Introduction

Neural mobilization (NM) is a physiotherapy procedure involving the passive mobilisation of limb nerve structures to restore normal movement and structural properties (1). Most research has been conducted using this technique in patients with chronic peripheral nerve pathologies who present with pain. Studies have shown a decrease in these symptoms in patients with cervical radiculopathy (2, 3), tension headache (4), cervical-brachialgia (5–7), sciatica (8), low back pain (9) and an increase in pain threshold in people with no pathology (10, 11). This technique also reduces pain caused by other pathologies that cause neuropathies, such as leprosy (12) or cancer (13), or due to other causes such as rheumatoid arthritis (14), osteoarthritis (15) and epicondylalgia (16).

Other effects of NM are increased ROM in subjects with (10, 17–20) and without pathology (3, 5, 21), changes in the muscular system, increasing strength (12, 15) and endurance (3), and increased recovery from fatigue (22). NM has also shown positive effects at a functional level by reducing disability in the affected limbs (3, 5, 6, 8, 12) and improving postural control in athletes (23). Finally, another phenomenon studied in cadavers has been the increased dispersion of intraneural fluid after the technique, which may be beneficial if intra-aneural edema is present (24, 25).

Even if these studies show that NM generates beneficial effects on different systems and subjects, research in humans does not allow us to describe which physiological mechanisms are involved in changes in pain perception and intensity, range of motion, and muscle strength, among others. To determine which systems are involved, it is necessary to look for basic research articles in which this technique has been used in animal models. In the literature, there are studies where physiotherapy techniques are applied to measure changes in biomarkers related to pain and inflammation and others to understand the processes involved in the changes produced by the techniques of this profession.

Suppose we use massage as an example and apply it to animal models. In this case, it has been studied to generate analgesic effects related to increased oxytocin in the periaqueductal gray matter (26) and changes in some genes that regulate inflammation (27). Another example that we can consider is passive joint mobilisations. They decrease pain by activating serotonergic and noradrenergic pathways (28, 29), whereas they decrease pro-inflammatory cytokines and increase anti-inflammatory cytokines (30, 31). The technique has also been studied to improve the repair of tissues such as bone (32) and cartilage (33, 34) by stimulating the tissues.

This systematic review aims to unify the information in the basic research literature on the physiological effects of NM on the peripheral and central nervous system (PNS and CNS) in animal models.

## 2 Methods

### 2.1 Search strategy

This review was performed following the guidelines of the Systematic Review Center for Laboratory Animal Experimentation (SYRCLE) (35) and the Preferred Reporting Items for Systematic Reviews and Meta-Analyses (PRISMA) principles (36). The protocol was registered in the International Prospective Register of Systematic Reviews (PROSPERO, CRD42022316225).

Two independent researchers performed the search (F.S. and L.M.-C.) in MEDLINE/PubMed and the Web of Science (WoS) between the 10th and 13th of February 2022 of articles published between January 1, 2012, and December 31, 2021. The search included the terms: “Neural mobilization OR Neurodynamics OR Nerve Mobilization” AND “Rats [MeSH] OR Mice [MeSH] OR Rabbits [MeSH] OR Cats [MeSH] OR Guinea Pigs [MeSH].” The search was limited to articles published only in English and performed in non-human species (other animals).

### 2.2 Study selection

For the study selection, two independent review authors (F.S. and L.M.-C.) screened titles and abstracts of retrieved documents to exclude irrelevant studies. After the duplicates were eliminated, abstracts were reviewed to identify eligible trials; at this stage, the inclusion and exclusion criteria were applied, and the selection was performed. Discrepancies between reviewers were resolved by discussion.

Studies meeting the following PICO criteria were selected: (i) Participants: rats, mice, rabbits, cats or guinea pigs with a model of neuropathic pain, (ii) Interventions: nerve mobilisation technique performed manually by an experimenter, (iii) Comparators: healthy controls and/or sham-operated animals, different intensity and frequency of interventions and (iv) Outcomes: central and/or peripheral biomarkers, pain-behavioral or other outcomes. The manuscripts selected included preclinical animal interventional studies.

Articles were excluded if they performed medical, veterinary studies, human studies or “*in vitro*” and “*ex vivo*” studies. They were also excluded if any drugs, invasive techniques, therapeutic exercise as the only therapy, or any bandages as treatment were applied. Systematic reviews, case reports and descriptive studies were excluded too. At least one control group had to be included.

### 2.3 Data extraction

Two independent reviewers (F.S. and L.M.-C.) extracted data from the selected studies. A third author was consulted in case of uncertainty (SV). The following data were extracted: (1) Authors and publication year, (2) Type of neuropathic pain model, (3) Groups, (4) Animal species, (5) Information about the intervention, (6) Outcomes.

### 2.4 Risk of bias and quality assessment

SYRCLE tool was used to analyse the risk of bias in each study (SV). This tool is based on Cochrane’s Risk of Bias tool for randomised clinical trials (RoB Tool) (37). It contains 10 items related to 6 types of bias (selection, performance, detection, attrition, reporting and others). The items were labelled as “yes” if they were free of risk of bias and “no” if they were not. When information was not reported, the risk of bias could not be discarded, and authors labelled the entry as “unclear.”

The methodological quality of the selected studies was evaluated by two independent authors (F.S. and S.V.) with the ARRIVE tool (38), consisting of 21 items to assess the reliability of the animal studies; the items were reported as “yes” (information included in the manuscript), “no” (information not included in the manuscript) or “X” (not applicable).

## 3 Results

### 3.1 Selection of the studies

**Figure 1:**
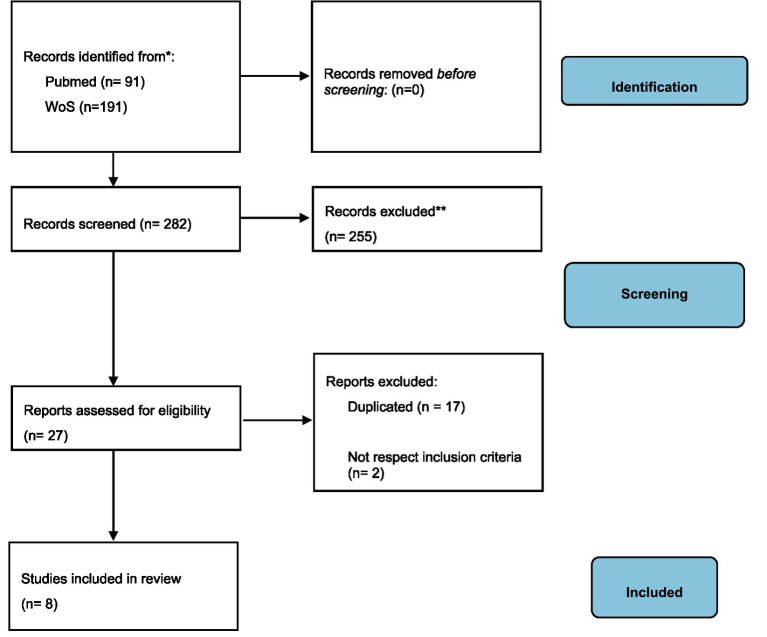
Flow Diagram PRISMA.

A total of 137 manuscripts were retrieved after the systematic search. After duplicate exclusion, 94 publications were assessed for eligibility. Of those, 85 were excluded because they did not meet the inclusion criteria. Finally, 8 articles were included in the systematic review. Figure 1 shows the flow diagram of the selection process.

### 3.2 Study characteristics

Table 1 shows the methodological characteristics of the studies. 7 articles performed preclinical studies of neuropathic pain in male Wistar and one in male Sprague–Dawley rats (39). 5 of them used a chronic constriction injury (CCI) model in the sciatic nerve. The CCI injury model was described by Bennet and Xie (40) and in brief, it consists of the exposure and ligation of the sciatic nerve (4 ligatures at a spacing of 1 mm) with a 4.0 chromic gut (41–45). In 2 studies (46, 47) the median nerve compression (MNC) protocol described by Chen et al. (48), performing 4 ligatures around the median nerve, was used. Only in one study (39) diabetic neuropathy (DN) induced by streptozocin injection was developed in Sprague–Dawley rats.

**Table 1:** Methodological characteristics of the selected studies.

Author, year	Neuropathic Pain model	Groups	Animal sp.	Treatment(s)	Measurements
Santos et al. (2012)	NP CCI	E1: (*n* = 6) CCI + NM E2: (*n* = 6) CCI S: (*n* = 6) Sham + NM N: (*n* = 6)	Male Wistar Rats	NM SN 2	NGF GFAP Mechanical Hyperalgesia Mechanical Allodynia Thermal Hyperalgesia
Marcioli et al. (2013)	NP MNC	E1: (*n* = 6) MNC only E2: (*n* = 6) MNC + 1 min NM E3: (*n* = 6) MNC + 3 min NM	Male Wistar Rats	NM MN	Mechanical Allodynia Fibre diameter Axon diameter Myelin sheath diameter
Santos et al. (2014)	NP CCI	E1: (*n* = 5) CCI + NM E2: (*n* = 5) CCI S1: (*n* = 5) Sham + NM S2: (*n* = 5) Sham N: (*n* = 5)	Male Wistar Rats	NM SN 2	DOR KOR MOR Sciatic Functional Index Muscle Function
da Silva et al. (2015)	NP CCI	E1: (*n* = 6) CCI + NM E2: (*n* = 6) CCI S: (*n* = 6) Sham + NM N: (*n* = 6)	Male Wistar Rats	NM SN 2	NGF MPZ Fibre diameter Axon diameter Myelin sheath diameter
Giardini et al. (2017)	NP CCI	E1: (*n* = 5) CCI + NM E2: (*n* = 5) CCI N: (*n* = 5)	Male Wistar Rats	NM SN 2	BDNF GFAP OX-42
Santos et al. (2018)	NP CCI	E1: (*n* = 10) CCI + NM E2: (*n* = 10) CCI S1: (*n* = 10) Sham + NM S2: (*n* = 10) Sham N: (*n* = 10)	Male Wistar Rats	NM SN 2	Substance P TRPV1 DOR KOR MOR
Marcioli et al. (2018)	NP MNC	E1: (*n* = 6) MNC only E2: (*n* = 6) MNC + 1 min NM E3: (*n* = 6) MNC + 3 min NM	Male Wistar Rats	NM MN	NGF BDNF
Zhu et al. (2018)	DN	E: (*n* = 6) S: (*n* = 6) N: (*n* = 6)	Male Sprague–Dawley	NM SN 2	TNFα IL-1β

BDNF, Brain-derived neurotropic factor; CCI, Chronic Constriction Injury; DN, Diabetic Neuropathy; DO, δ-opioid receptor; E, Experimental; GFAP, Glial Fibrillary Acidic Protein; IL-1β, Interleukin 1 beta; KOR, κ-opioid receptor; MNC, Median Nerve Compression; MOR, μ-opioid receptor; MPZ, Myelin Protein Zero; N, Naive; NGF, Neural Growth Factor; NM, Neural Mobilization; OX-42, Microglial Cell OX-42; S, Sham; SFI, Sciatic Functional Index; TNFα, Tumor Necrosis Factor Alpha; TRPV1, Transient Receptor Potential Vanilloid 1.

All the studies included at least one control group. In those cases where the CCI injury was performed, a sham group (nerve exposure without compression) and a naive group were used as controls (41–45). The MNC protocol without NM served as a control in the articles of Marcioli et al. (46, 47) and the diabetic neuropathy model included a sham (saline injection) and a naïve group (39). The sample size varied from 5 to 10 animals per group. A total of 192 animals were included in the studies.

Regarding the interventions, the NM protocol started 3 (46, 47, 49), 10 (39) or 14 (41–45) days after the neuropathic induction and consisted of repeated oscillations of the scapular limb (46, 47) or the ankle joint (39, 41–45, 49). The mobilisations were performed in a wide range of time, from 1 or 3 min (46, 47) to 10 min (39, 41–45, 49) and the duration of the treatment lasted from 10 days (41–47, 49) to 3 weeks (39).

### 3.3 Risk of bias and reporting quality

All the selected articles were assessed for six different types of risks of bias using SYRCLE tool Table 2. When information was reported, most of the studies (41, 43–46, 49) were free of selection bias, whereas one (39) was not for showing differences between animal groups at the baseline. None of the studies reported information about allocation generation and concealment. Concerning performance bias, none of the studies reported random housing, so those whose dependent variables could be affected by light or room temperature (pain, mostly) were not considered free of performance bias (39, 41, 46, 49). However, the rest of the articles were not performance-biased (42–47). Similarly, although investigator blinding was not reported in any study, outcome assessment was considered free of detection bias in most cases (39, 41, 42, 45–47, 49) since computer-based techniques were used. The attrition bias could not be studied since most of the studies did not report information about it, except for two studies (39, 41) which were not biased. Only one did show unexplained missing data and was considered likely to have attrition bias (45). When reported, all the articles but one (47) were free of reporting bias due to the use of thorough study protocols. Lastly, the authors considered 5 studies bias-free for other reasons (39, 41, 42, 44, 45). Only 3 studies (43, 46, 47) did show some concerns, mainly about study design or lack of data dispersion. In summary, most of the studies (41, 43–45, 47, 49) only showed one risk of bias, or two (39, 46) and (42) were free of any analysed bias when enough information was reported.

**Table 2:** Risks of bias analysis of the selected studies using the SYRCLE tool.

	Selection bias			Performance bias		Detection bias		Attrition bias	Reporting bias	Other
Study	1. Sequence generation	2. Baseline characteristics	3. Allocation concealment	4. Random housing	5. Researchers or caregivers blinding	6. Random outcome assessment	7. Assessors blinding	8. Incomplete outcome reporting	9. Selective outcome reporting	10. Other sources of bias
Santos et al. (2012)	Unclear	Yes	Unclear	No	Unclear	Unclear	Yes	Yes	Yes	Yes
Marcioli et al. (2013)	Unclear	Yes	Unclear	No	Unclear	Unclear	Yes	Unclear	Yes	No
Santos et al. (2014)	Unclear	Unclear	Unclear	Yes	Unclear	Unclear	Yes	Unclear	Yes	Yes
da Silva et al. (2015)	Unclear	Yes	Unclear	Yes	Unclear	Unclear	Unclear	Unclear	Yes	No
Giardini et al. (2017)	Unclear	Yes	Unclear	Yes	Unclear	Unclear	Unclear	Unclear	No	Yes
Santos et al. (2018)	Unclear	Yes	Unclear	Yes	Unclear	Unclear	Yes	No	Yes	Yes
Marcioli et al. (2018)	Unclear	Unclear	Unclear	Yes	Unclear	Unclear	Yes	Unclear	Unclear	No
Zhu et al. (2018)	Unclear	No	Unclear	No	Unclear	Unclear	Yes	Yes	Yes	Yes

Items are labelled “yes” when are free of risk of bias, “no” when they are not, and “unclear” when information was not reported, and risk of bias cannot be discarded.

Table 3 summarises the reporting quality of the selected studies using the updated ARRIVE tool. In agreement with SYRCLE tool, there was a significant lack of information for several items (labelled as “no”). None of the studies reported information about allocation randomisation, investigators blinding or describing protocols to reduce suffering. Remarkably, experimental procedures were reported in all cases, providing detailed information about time, frequency and reasons for intervention. Moreover, in all cases, the background was enough and coherent, and the objectives and rationale were well scientifically explained. Also, animal handling and wellness are maintained across the studies as required in animal research to preserve their welfare and prevent or reduce their suffering during studies (ethics and husbandry).

**Table 3:** Quality report of the selected studies using the updated ARRIVE tool.

	1. Study design		2. Sample size		3. Inclusion and exclusion criteria			4. Randomisation		5. Blinding	6. Outcome measures		7. Statistical methods		8. Experimental animals		9. Experimental procedures				10. Results	
	1.a	1.b	2.a	2.b	3.a	3.b	3.c	4.a	4.b	5.a	6.a	6.b	7.a	7.b	8.a	8.b	9.a	9.b	9.c	9.d	10.a	10.b
Santos et al. (2012)	Yes	Yes	Yes	No	No	No	Yes	No	No	No	Yes	No	No	No	Yes	No	Yes	Yes	Yes	Yes	Yes	x
Marcioli et al. (2013)	Yes	Yes	No	No	No	No	Yes	No	No	No	Yes	No	No	Yes	No	No	Yes	Yes	Yes	Yes	Yes	x
Santos et al. (2014)	Yes	Yes	No	No	No	No	Yes	No	No	No	Yes	No	Yes	No	Yes	No	Yes	Yes	Yes	Yes	Yes	x
da Silva et al. (2015)	Yes	Yes	No	No	No	No	Yes	No	No	No	Yes	No	Yes	No	Yes	No	Yes	Yes	Yes	Yes	No	x
Giardini et al. (2017)	Yes	Yes	No	No	No	No	Yes	No	No	No	Yes	No	Yes	No	Yes	No	Yes	Yes	Yes	Yes	Yes	x
Santos et al. (2018)	Yes	Yes	No	No	No	No	Yes	No	No	No	Yes	No	Yes	No	No	No	Yes	Yes	Yes	Yes	Yes	x
Marcioli et al. (2018)	No	Yes	No	No	No	No	Yes	No	No	No	Yes	No	Yes	No	Yes	No	Yes	Yes	Yes	Yes	Yes	x
Zhu et al. (2018)	Yes	Yes	Yes	No	Yes	No	Yes	No	No	No	Yes	No	Yes	No	No	No	Yes	Yes	Yes	Yes	Yes	x

	11. Abstract	12. Background		13. Objectives	14. Ethical statement	15. Housing and husbandry	16. Animal care and monitoring			17. Interpretation/ scientific implications		18. Generalisability/ translation	19. Protocol registration	20. Data access	21. Declaration of interests	
	11. a	12. a	12. b	13. a	14. a	15. a	16. a	16. b	16. c	17. a	17. b	18. a	19. a	20. a	21. a	21. b
Santos et al. (2012)	Yes	Yes	Yes	Yes	Yes	Yes	No	No	No	Yes	No	Yes	No	Yes	Yes	No
Marcioli et al. (2013)	Yes	Yes	Yes	Yes	Yes	Yes	No	No	No	Yes	No	Yes	No	Yes	Yes	No
Santos et al. (2014)	Yes	Yes	Yes	Yes	Yes	Yes	No	No	No	Yes	No	Yes	No	Yes	Yes	No
da Silva et al. (2015)	Yes	Yes	Yes	Yes	Yes	Yes	No	No	No	Yes	No	No	No	Yes	Yes	No
Giardini et al. (2017)	yes	yes	no	yes	yes	yes	no	no	no	yes	no	yes	no	yes	yes	no
Santos et al. (2018)	Yes	Yes	Yes	Yes	Yes	Yes	No	No	No	Yes	No	Yes	No	Yes	Yes	Yes
Marcioli et al. (2018)	No	Yes	Yes	Yes	Yes	Yes	No	No	No	Yes	Yes	No	No	Yes	Yes	No
Zhu et al. (2018)	Yes	Yes	Yes	Yes	Yes	Yes	No	No	No	Yes	Yes	Yes	No	Yes	Yes	No

Items are labelled “yes” when requirements are met by the publication, “no” when they are not and “x” if question is not applicable to the study.

### 3.4 Main outcomes and results

Table 4 shows the main results of the studies included in the review. Among the selected studies, only two performed NM on the upper limbs (46, 47) and the rest on the lower limbs (39, 41–45, 49).

**Table 4:** Results of the selected studies.

Author	Peripheral Biomarkers	Central Biomarkers	Pain-Behavioral Outcomes	Others
Santos et al. (2012)	*DRG* ↓ NGF ↓ GFAP	*SC* NGF: No differences ↓ GFAP	↓ from 2nd session: Mechanical Hyperalgesia Mechanical Allodynia Thermal Hyperalgesia	─
Marcioli et al. (2013)	_	_	Mechanical Allodynia: No differences	No differences: Fibre diameter Axon diameter Myelin sheath diameter
Santos et al. (2014)	_	*PAG* ↑ DOR ↑ KOR MOR: No differences	─	↑ SFI ↑ Muscle Function
da Silva et al. (2015)	*Nerve* ↑ NGF ↑ MPZ	_	─	↑ Fibre diameter ↑Axon diameter ↑ Myelin sheath diameter
Giardini et al. (2017)	_	*Thalamus and PAG* ↓ BDNF ↓ GFAP ↓ OX-42	─	─
Santos et al. (2018)	*DRG* ↓Substance P ↓TRPV1 ↑ MOR DOR: No differences KOR: No differences	_	─	─
Marcioli et al. (2018)	*Nerve* NGF: No differences BDNF: No differences	_	─	─
Zhu et al. (2018)	*Nerve* ↓ TNFα ↓ IL-1β	_	↓ Mechanical Allodynia	─

BDNF, Brain-derived neurotropic factor; DOR, δ-opioid receptor; DRG, Dorsal Root Ganglion; GFAP, Glial Fibrillary Acidic Protein; IL-1β, Interleukin 1 beta; KOR, κ-opioid receptor; MOR, μ-opioid receptor; MPZ, Myelin Protein Zero; NGF, Neural Growth Factor; OX-42, Microglial Cell OX-42; PAG, Periaqueductal Gray; SC, Spinal Cord; SFI, Sciatic Functional Index; TNFα, Tumor Necrosis Factor Alpha; TRPV1, Transient Receptor Potential Vanilloid 1.

The NM treatment induced changes in biomarkers at both central and peripheral levels when applied at least 10 days after the neuropathic pain induction protocol. Thus, in the CCI injury model, there was a decrease in Neural Growth Factor (NGF), Glial Fibrillary Acidic Protein (GFAP), substance P and Transient Receptor Potential Vanilloid 1 (TRPV1) and an increase of μ-opioid receptor (MOR) in dorsal root ganglion (39, 41, 45) after NM. In contrast, in the peripheral nerve, an increase of NGF and Myelin Protein Zero (MPZ) (43) and a decrease of Tumor Necrosis Factor Alpha (TNFα) and Interleukin 1 beta (IL-1β) (39) were shown. Others opioid receptors, DOR and KOR did not suffer changes in the dorsal root ganglion (45). Significant changes were found in the spinal cord, where there was a decrease in NGF levels (41) and periaqueductal grey and thalamus, showing an increase in opioid receptors DOR and KOR (42) and a decrease of Brain-derived neurotrophic factor (BNDF), GFAP and markers of Microglial Cell OX-42 (OX-42) levels (44). On the contrary, when the NM was applied only 3 days after the neuropathy induction, it did not change the peripheral nerve’s NGF and BDNF (47).

The same pattern was observed regarding pain-behavioral outcomes: both mechanical and thermal hyperalgesia and allodynia decreased when the nerve mobilisation was applied at least 10 days after the neuropathy induction (39, 41), but no differences with the control group were found in mechanical allodynia in the study of Marcioli et al. (46), where the mobilisation protocol started only 3 days after surgery.

The fibre diameter, axon diameter and myelin sheath diameter of the injured nerves were studied by Marcioli et al. (46), who did not find changes using the nerve mobilisation protocol applied 3 days after surgery, while da Silva et al. (43) found an increase in axon, sheath and myelin diameters when the NM protocol was applied 14 days after the CCI was performed.

## 4 Discussion

This review gathered information on the physiological effects of NM on the PNS and CNS. Our results show that there is medium to high evidence, considering that all studies were RCTs, but it has not been possible to perform a meta-analysis of the results.

Concerning bias and quality of studies, none of the studies provided information about allocation concealment and researchers’ blinding. Implementation of tools preventing the risk of bias.

According to the results of this systematic review, inflammation decreases when NM is applied 10 days after CCI, as shown by the reduction of most inflammatory molecules. GFAP is a marker of activated satellite glial cells in PNS, which increases after CCI and nerve ligation in rodents (50, 51). Substance P, IL-1β and TRPV1 also contribute to inflammatory responses related to rodent neuropathic pain (52–55). NGF appears to mediate chronic neuropathic pain and increases after CCI in rodents (56). However, its levels are reduced after NM only in the DRG and spinal cord, whereas the peripheral nerve remains unchanged.

Moreover, the increase in MPZ may be related to Schwann cell regeneration, nerve recovery, and remyelination. An increase in opioid receptors has been linked to higher tolerance to pain (57). Thus, the increase of MOR in the PNS observed after NM, along with the reduction of the inflammatory mediators listed above, may contribute to the analgesic effects of NM reported in the studies of this systematic review. These effects have not been found if NM was performed only 3 days after surgery, probably because the effects are not the same if it is an acute pathology phase.

Animal models of neuropathic pain have limitations, and their translation to humans is complex. However, it is well described how nerve damage-induced release of inflammatory mediators sensitises and activates nociceptors and contributes to chronic pain (58). Therefore, we hypothesized that the NM induces analgesia observed in patients (2–16), which may be mediated by the reduction of inflammatory mediators by glial cells and the activation of the opioid system.

The information found in this study agrees with a recent review of the effects of other physical therapy techniques on biomarkers related to neuropathic pain (59). The authors conclude that most physiotherapeutic interventions modulate the expression of molecular mediators of pain.

Finally, although NM has been shown to cause changes in the muscular system in humans (3, 12, 15, 22), no studies in animal models have examined the physiological mechanisms involved. This could be an area of research for future studies.

## 5 Conclusion

We conclude that NM changes the peripheral and central nervous systems of animal models. The physiological changes studied are related to pain modulation and include the endogenous opioid analgesic system at both the central and peripheral levels and inflammatory modulators at the central and peripheral levels. Another system involved in both the PNS and CNS is the immune system, which relates to and modulates the other two systems, analgesic and inflammatory. We suggest further investigation and focus on improving the quality of the studies following the ARRIVE criteria and finding if more systems are involved in the changes in muscles and other structures.

## 6 Limitations

There are some limitations to be considered when reading this study. First, we included only studies in English. Regarding the quality of the studies, none described the randomization of groups, blinding of investigators, or protocols to reduce animal suffering. Another significant limitation was that we could not perform a meta-analysis because the articles did not study the same outcomes. Finally, more research is needed to transfer the effects studied in these animal trials to humans.

## Data availability statement

The original contributions presented in the study are included in the article/Supplementary material, further inquiries can be directed to the corresponding author.

## Author contributions

FS: Conceptualization, Data curation, Formal analysis, Funding acquisition, Investigation, Methodology, Writing – original draft, Writing – review & editing. SV: Data curation, Formal analysis, Methodology, Supervision, Writing – review & editing, Writing – original draft. LM-C: Conceptualization, Methodology, Writing – original draft.
