# Supplementary material for: Peripheral and central changes induced by neural mobilization in animal models of neuropathic pain: a systematic review
Source: Front Neurol. 2024 Jan 5;14:1289361. doi: 10.3389/fneur.2023.1289361 (PMC10797109; doi:10.3389/fneur.2023.1289361)
Supplement: Supplementary file 1 [file Data_Sheet_1.PDF]

Supplementary Material

1 Supplementary Data

The search included the following terms and filters for all the databases:

Filters:

Year of publication: January 1, 2012 - December 31, 2021

Species: Other Animals

Article Language: English

Terms

Neural mobilization AND Rats [MeSH]

Neural mobilization AND Mice [MeSH]

Neural mobilization AND Rabbits [MeSH]

Neural mobilization AND Cats [MeSH]

Neural mobilization AND Guinea Pigs [MeSH]

Neurodynamics AND Rats [MeSH]

Neurodynamics AND Mice [MeSH]

Neurodynamics AND Rabbits [MeSH]

Neurodynamics AND Cats [MeSH]

Neurodynamics AND Guinea Pigs [MeSH]

Nerve Mobilization AND Rats [MeSH]

Nerve Mobilization AND Mice [MeSH]

Nerve Mobilization AND Rabbits [MeSH]

Nerve Mobilization AND Cats [MeSH]

Nerve Mobilization AND Guinea Pigs [MeSH]
